# Optimization of Crumb Rubber Modified Binder Formulation through Compatibility Analysis

**DOI:** 10.3390/ma16155357

**Published:** 2023-07-30

**Authors:** Svetlana Obukhova, Evgenii Korolev, Angelina Budkina

**Affiliations:** 1 Department of Urban Planning, Institute of Architecture and Urban Planning, National Research Moscow State University of Civil Engineering, Moscow 129337, Russia; angelina-line@yandex.ru; 2 Scientific and Educational Center “Nanomaterials and Nanotechnologies”, National Research Moscow State University of Civil Engineering, Moscow 129337, Russia; korolev@nocnt.ru

**Keywords:** crumb rubber, bitumen, crumb rubber modified bitumen, structure, compatibility, stability

## Abstract

The research is devoted to developing the production of crumb rubber-modified bitumen with improved stability. It has been established that the most suitable semi-empirical coefficient for determining the compatible plasticizer to crumb rubber is based on the ratio of paraffin-naphthenic compounds to resinous-asphaltene compounds. With the help of differential scanning calorimetry, temperature regimes of crumb rubber destruction and preparation of rubber-containing components (210 °C) were studied and determined. It was established that determining the dynamic viscosity of hydrocarbon concentrates with crumb rubber on a rotary viscometer is not applicable due to elastic components, making it difficult to measure and obtain reliable data. The most suitable method is the shear viscosity method. Using fluorescent microscopy, it was established that the formation of a branched structure of crumb rubber is achieved in the waste industrial oil, indicating devulcanization processes. It was found that hydrocarbon plasticizer with high naphthenic oil content is the most compatible with crumb rubber. Synthetic wax was found to be of greater interest as a devulcanizing/stabilizing agent, and its application in an amount of 3% allows the formation of a stable CRMB structure and stabilizes the devulcanization process.

## 1. Introduction

The task of improving the safety and durability of road surfaces remains both scientifically and practically relevant. An independent way of modifying petroleum bitumen is by injecting crumb rubber obtained from the complex processing of end-of-life tyres and other rubber products [1,2,3]. This method contains the obvious advantages [4,5] and negative experience of operating asphalt concrete modified with crumb rubber [6,7]. Several promising methods have been developed to eliminate the disadvantages of bitumen modification with crumb rubber, which usually consists of the destruction of the rubber surface due to strong shear effects [8,9,10], the impact of ionizing radiation [11] or devulcanizer treatment [12,13,14,15]. However, for these methods, the problem of dispersion in the bitumen remains due to a significant increase in the surface area of the rubber modifier and/or a change in the wettability of the crumb rubber surface. This leads to a lack of homogeneous distribution of crumb rubber and the formation of aggregates consisting of non-wetted particles—an elastic after effect which causes intensive cracking, especially in the low-temperature operation period [16,17]. In this regard, the world community is developing other ways of modifying bitumen with crumb rubber.

Based on a review of scientific and technical literature, it was found that to achieve a uniform devulcanization of rubber and improve the properties of crumb rubber modified bitumen (CRMB), crumb rubber with a specific morphology must be selected. Thus, the maximum to a minimum particle size of crumb rubber should be the smallest, and the crumb rubber should not exceed 1–3 mm [3]. The necessity of pre-treatment crumb rubber in plasticizers, which should be characterized by a high ratio of paraffin-naphthenic compounds, asphaltenes and generally lower viscosity, has been established [18,19]. The common problem is ensuring effective dispersion and uniform volume distribution of modifiers in the hydrocarbon carrier and in the bitumen binder matrix to be modified [20,21].

**Figure 1:**
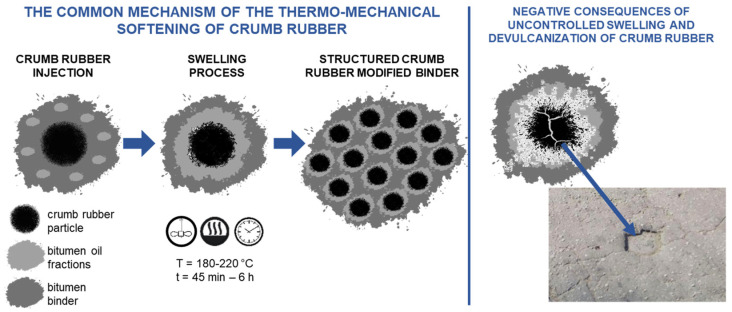
Mechanism of thermo-mechanical plasticization of crumb rubber.

Analysis of the data and hypotheses presented by the authors of various publications [9,20,22,23,24,25,26] shows that there is currently no unified scientific knowledge about the physical and chemical mechanisms leading to producing rubber-bitumen binders with high-performance properties. In most cases, the authors describe the process of thermo-mechanical plasticization of crumb rubber as follows: during the joint thermo-mechanical treatment, the crumb rubber swells in the oil fractions of bitumen, which weakens the inter-molecular bonds in the rubber. Because of continuing heat and mechanical influences, these weakened bonds are broken, i.e., the destruction/devulcanization of rubber with the formation of rubber substance, which diffuses into the bitumen and structures it [27,28]. The mechanism of thermo-mechanical plasticization of crumb rubber is shown schematically in Figure 1. The structuring function of the rubber substance explains the effects of increasing the deformation stability of rubber-bitumen binder and asphalt concrete on its basis.

However, it should be noted that the lack of knowledge about the features of the structure formation process during rubber devulcanization and modification of bitumen leads to variability of the research results and ambiguity of the recommended technological parameters. So, if not stabilized and stopped the devulcanization process, the crumb rubber continues swelling in bitumen, which will destroy the crumb rubber. And pre-formed rubber structuring centers will be destroyed. And the incoherent particles of crumbs rubber will be chipping on the road's surface and leading to the destruction of the road. No scientifically grounded criteria exist for obtaining effective, stable dispersion systems containing crumb rubber. The lack of knowledge about the interaction process of liquid hydrocarbon carriers and devulcanized crumb rubber does not allow effective control of the dissolution process to obtain dispersion systems with given parameters of properties and their stability.

This research is devoted to developing a scientifically justified technological solution for producing CRMB with improved performance properties. Implementing the scientifically substantiated technological solution will increase the durability of asphalt concrete, reduce the cost of road construction, and increase its service life.

The phase compositions of crumb rubber and hydrocarbon plasticizers are complex, and molecules in different phase states exhibit other mixing behaviour. Therefore, to predict the compatibility of the hydrocarbon plasticizers and to be able to optimise the experiment, let us consider existing semi-empirical compatibility parameters.

1. The Traxler dispersion coefficient [29] considers the ratio between the sum of resins and aromatic hydrocarbons and the sum of asphaltenes and paraffin-naphthenic hydrocarbons:

(1) $$C_{D}=\frac{Ar+R}{PN+As\;},$$

where *C_D_*—is the Traxler dispersion coefficient; *Ar*—is the number of aromatic hydrocarbons; *R*—the number of resins; *PN*—the number of paraffin-naphthenic hydrocarbons; *As*—the number of asphaltenes.

The higher the Traxler dispersion coefficient value, the more aromatic fractions and resins in the plasticizer have a high affinity to the crumb rubber. They can dissolve the polymer molecules of the rubber [30].

2. The mass ratio of paraffin-naphthenic compounds to asphaltenes (*PN*/*As*) indirectly characterizes the system's viscosity, which affects the rate of swelling and dispersion of rubber molecules. The higher the *PN*/*As* ratio, the better solubility and lower viscosity of the plasticizer and the faster swelling and dispersing processes of rubber molecules [31].

3. The Hildebrand solubility parameter [32] is used to predict the solubility of a polymer (modifier) in various organic solvents. This parameter characterizes the intensity of intermolecular interactions in the substance. It is numerically equal to the energy expended in removing the molecules to an infinitely large distance (at which the interaction forces can be neglected). The solubility parameter is calculated using the following formula:

(2) $$\delta_{P}=\sqrt{\frac{E}{V}},$$

where *δ_P_*—is the solubility parameter of the plasticizer; *E*—the evaporation energy; *V*—the volume of the substance. These concepts are extended to organic solvents and polymers, with estimates given per repeating link of the polymer. The difficulty here is that *δ* can only be determined experimentally for low molecular weight liquids that evaporate without decomposition. For polymers and plasticizers with complex hydrocarbon compositions that cannot be vaporized without decomposition, *δ* values are determined by indirect methods or calculation. To determine the Hildebrand solubility parameter for organic plasticizers using the indirect method, the same volume of liquid was taken to determine the calculated parameters.

Troughton’s equation is applicable to determine the evaporation energy of organic substances:

(3) $$E=kT_{B},$$

where *k*—a constant equal to 89.2 J/(mol*K); *T_B_*—the boiling point.

Thus, to determine the compatibility of the plasticizer and modifier, the following condition must be fulfilled:

(4) $$\delta_{P}=\sqrt{\frac{E}{V}}=\sqrt{\frac{kT_{F}}{V}}\approx \delta_{M}=\sqrt{\frac{E}{V}}=\sqrt{\frac{kT_{BO}}{V}},$$

where *δ_P_*, *δ_M_*—solubility parameters of plasticizer and modifier respectively; *E*—evaporation energy; *V*—substance volume; *k*—a constant equal to 89.2 J/(mol*K); *T_B_*—boiling temperature; *T_F_*—flashing temperature of plasticizer; *T_BO_*—burnout temperature of modifier.

Thus, the formation of stable dispersion systems containing crumb rubber (modifier) with the considered hydrocarbon plasticizers will become possible only when the parameter Δ*δ = δ_P_ − δ_M_* tends to 0.

The existing methods of modifying bitumen are aimed at stabilizing its component composition both in the initial period of structure formation and in the operational period. The developed methods are quite effective but require the involvement of complex synthetic substances. At the same time, the critical parameters of CRMB structure formation are not only the formation of the polymer net spread in the bitumen volume, with nodes of rubber particles, but the thickness of the transition zone formed by rubber and bitumen devulcanization products. The thickness of this transition layer will determine the stability of the structure and the performance properties of the modified bitumen, both in the initial period and during operation. The solution to this problem involves solving several issues, including the devulcanization of crumb rubber. The general principle of the devulcanization process is known; it consists of partial destruction of the rubber, resulting in local destruction of its spatial structure. Rupture of the spatial mesh during devulcanization occurs both at the sulfur attachment point and in the main molecular chains. The spatial structure of the vulcanizate “loosens”. That is, the density of the spatial network decreases due to the breakdown of some transverse bonds and some of the main molecular chains, which leads to a soluble fraction with an average molecular weight of 6000–12,000. Devulcanization can be initiated by mechanical, thermal, and chemical energy or a combination thereof. An important factor here is the presence of an additional component that can reduce the energy input for the spatial opening of the system. Another important issue is the uncontrollability of the devulcanization process, which can lead to an unstable final composite. Paraffin can act as an inhibitor and stabilizer of this process, which molecules, when introduced at the final stage of preparation, will envelop the swollen particle of crumb rubber, and form a transitional shell, which prevents its subsequent adsorption of oils from the bitumen binder. And contribute to a more uniform distribution of crumb rubber in the volume of the composite. In this way, a controlled devulcanization process can be achieved.

Construction materials science actively attracts the results of fundamental research to develop new science-based technological solutions. It obtains new knowledge by identifying previously unknown facts of the joint influence of various controlling factors. The accumulated experience of various methods and approaches to bitumen modification and using crumb rubber in road construction indicates the need to find such a synergistic system and optimise crumb rubber-modified binder formulation through compatibility analysis. This is a new methodological approach to the development of technological solutions.

## 2. Materials and Methods

### 2.1. Raw Materials and Characterization

Crumb rubber (CR 0.5) is obtained by crushing and grinding waste rubber and technical products—pneumatic tires of passenger vehicles. With the gradual removal of textile, synthetic and metal cords. Manufactured by LLC Chekhov Regeneration Plant, Chekhov, Russia. Parameters of the physical properties of investigated crumb rubber are presented in Table 1.

Hydrocarbon plasticizers were considered liquid hydrocarbon carriers:

1. Medium-viscous petroleum residual extract produced by LLC “LUKOIL-Volgogradneftepererabotka”, Volgograd, Russia. The technology of the residual extract production is the following: tar from the vacuum part (without oxygen access) of the atmospheric-vacuum column goes to propane deasphaltization, where it is separated into asphalt and deasphaltizate. Then the deasphaltizate goes to selective purification, where it is separated into a refinery, from which diesel fuel is produced, and a petroleum residual extract. Physicochemical properties are presented in Table 2.
2. Waste industrial oil after use in the unit of the ammonia production shop. Waste industrial oil was provided by the Azot Branch of URALCHEM, JSC, in Berezniki, Perm Region, Russia. The physical and chemical properties are presented in Table 3.

The devulcanizing agents considered were:

1. Poly-transoctenamer rubber (TOR), which is produced based on cyclooctene and has a high proportion of trans-double bonds, is produced in Germany;
2. Synthetic wax obtained by Fischer-Tropsch synthesis from natural gas in special reactors produced in Russia.

For the preparation of CRMB oil road bitumen grade BND 50/70, produced by LLC “LUKOIL-Nizhegorodnefteorgsintez” (Kstovo, Russia) was used. Bitumen was tested for compliance with the requirements of Interstate Standard GOST 33133-2014 [33]. The results of laboratory tests of the physical and mechanical properties of bitumen are shown in Table 4.

### 2.2. Method for Determination of Group Hydrocarbon Composition of Plasticizers

**Figure 2:**
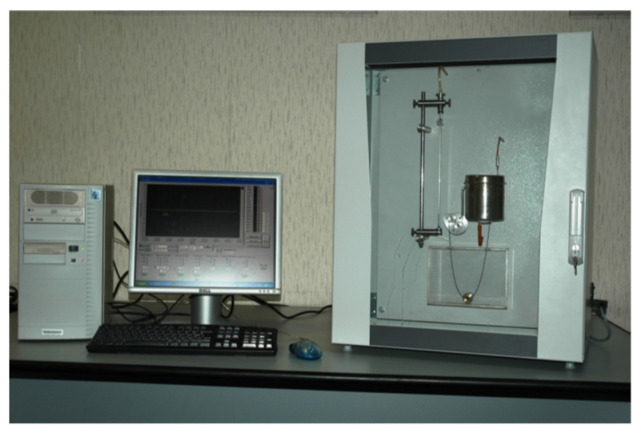
**“**Gradient M” unit designed by SUE INHP RB.

For the calculation of semi-empirical parameters, the group hydrocarbon composition of plasticizers was determined by liquid adsorption chromatography with gradient displacement on the laboratory unit “Gradient M” by SUE INHP RB. Installation “Gradient M” (Figure 2) is designed for the quantitative determination of the hydrocarbon composition of heavy oil fractions—oils, vacuum gas oil, fuel oils, tar, cracking residuals, oxidized and natural bitumen. The essence of the method consists of a stepwise gradient-displacement separation of high-boiling heavy oil products into seven groups, followed by their registration with a thermal conductivity detector.

The principle of operation of the “Gradient-M” unit is as follows:

- the separation of the analysed product in the chromatographic column by a mobile phase flow consisting of a solvent mixture selected for a particular separation case;
- the transfer of the eluent in the form of a film on the transport chain;
- the removal of mobile phase components in the evaporator;
- the thermal oxidative degradation of separated components of the analysed substance in the oxidation cell;
- the detection of the formed carbon dioxide by the thermal conductivity detector.

**Figure 3:**
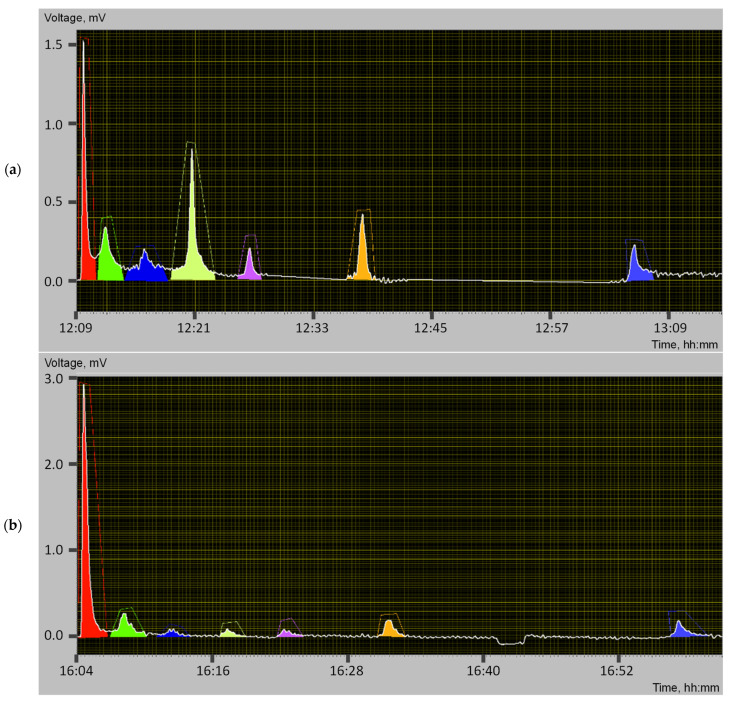
Chromatograms: (**a**) Petroleum residual extract; (**b**) Waste industrial oil.

The separated components are fed to the conveyor chain through a needle that ends the column. The eluent is removed from the chain as it moves through the evaporator. The mixture components enter the oxidation cell, where they are transformed at high temperatures in the presence of air oxygen and copper oxide into carbon dioxide, which is detected in the catarometer. The catarometer compares the thermal conductivity of pure air and air and carbon dioxide mixtures. This difference in thermal conductivity leads to an unbalanced equilibrium detector bridge. The recording of the detector signals on the monitor screen is a chromatogram, with each mixture group corresponding to a specific peak, Figure 3. Three measurement samples were taken from each plasticizer. The standard deviation for all samples was not more than 4%.

### 2.3. Differential Scanning Calorimetry of Crumb Rubber

The mixing temperature of the “hydrocarbon plasticizer—crumb rubber” dispersion system was determined by thermal analysis using a LINSEIS DSC PT-1600 (Linseis GmbH, Selb, Germany) —high high-temperature differential scanning calorimeter. The crumb rubber sample was subjected to differential scanning calorimetry (DSC). A thermo-analytical technique in which the difference in the amount of heat required to raise the temperature of the sample and the standard is measured as a function of temperature. The main property measured with DSC is the heat flux, the flow of energy into/from the sample as a function of temperature or time, usually shown in units of mJ/s on the y-axis. In DSC, thermal changes occurring in the rubber particles result in the absorption (endothermic process) or release (exothermic process) of heat. Endothermic changes include evaporation, phase changes such as melting, sublimation, transition between two different crystalline structures, decomposition, etc.

In contrast, exothermic changes include crystallization, chemisorption, oxidation-reduction, etc. Thus, any change of state can be detected by measuring the temperature difference. Two samples were taken from crumb rubber for research. The standard deviation was not more than 5%.

### 2.4. Methods for the Preparation and Characterization of Dispersion Systems

**Figure 4:**
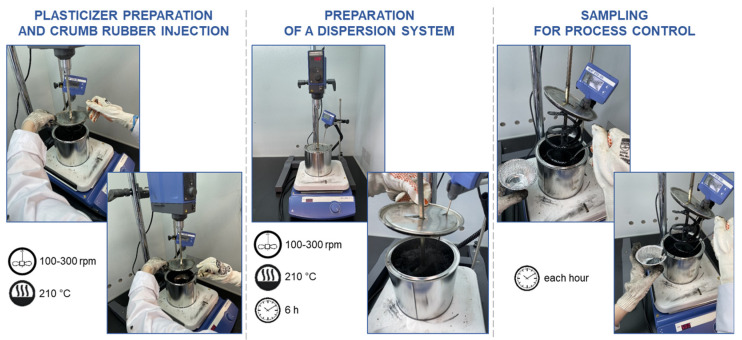
Schematic illustration of the methodology for the preparation of “hydrocarbon plasticizer—crumb rubber” dispersion systems.

The methodology for the “hydrocarbon plasticizer—crumb rubber” dispersion system is as follows. The required plasticizer and crumb rubber amount is weighed in the first step. The plasticizer is then poured into a container with a sealed lid into which a mixer and a heat control sensor are immersed. The mixer is turned on at 100–300 rpm, and the dispersion system is heated to 210 °C. Then at the second stage, when the set temperature is reached in a container with a plasticizer at a stirring speed of 300 rpm, crumb rubber is gradually introduced for 10–15 min. The container is hermetically sealed, and the devulcanization process starts, lasting no longer than 6 h. Samples are taken every hour. The methodology for the “hydrocarbon plasticizer—crumb rubber” dispersion system is shown schematically in Figure 4.

The devulcanization process of crumb rubber, accompanied by an increase in its initial volume (swelling), was studied by determining the dispersion system's hydrocarbon group composition, dynamic viscosity, and shear viscosity during preparation. A Rheolab QC rotary rheometer with a controlled shear rate based on the Searle principle of rotating concentric cylinders was used to determine the dynamic viscosity. The determination of dynamic viscosity was carried out using a coaxial cylinder measuring system. A DSR dynamic shear rheometer based on the principle of adjustable shear strain was used to determine shear viscosity to measure flow properties. The determination of shear viscosity was carried out using a geometry (two discs) where the pad diameter was 25 mm. On the rotational rheometer, the test temperature was 135 °C, and the shear rate was 30 s^−1^. On a dynamic rheometer, the test temperature is similar—135 °C, G*/sin *δ* ≥ 1 kPa. At least three samples are prepared and tested for each percentage of crumb rubber. The standard deviation was not more than 4%.

The uniform distribution of the crumb rubber in the volume of the hydrocarbon plasticizer was assessed by fluorescence microscopy on the MIKMED-2 Luminescence Microscope instrument (LLC “Leningrad Optical-Mechanical Association”, Saint Petersburg, Russia).

### 2.5. Methods for the Preparation and Characterization of CRMB

**Figure 5:**
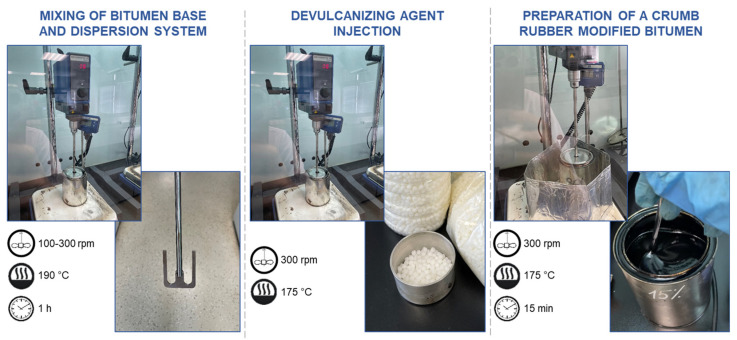
Schematic illustration of the methodology for the preparation of CRMB.

The methodology for the preparation of CRMB is as follows. In the first stage, the necessary amount of the “hydrocarbon plasticizer—crumb rubber” dispersion system and bitumen base are weighed. The components are then poured into a container with a sealed lid, where an anchor-type mixer and a heat control sensor are immersed. The mixer is started at 100–300 rpm, and the system is preheated to 190 °C. Once 190 °C is reached, the system components are stirred at a mixing speed of 300 rpm for one hour. In the second step, the system's temperature is reduced to 175 °C and the devulcanizing agent is gradually introduced at a stirring speed of 300 rpm over 2–3 min. The container is then closed, and the system with the devulcanizing agent is stirred for 15 min at 175 °C and a stirring speed of 300 rpm. At the end of the preparation of the rubber asphalt binder, the container is removed from the heating plate, and the system is cooled down to room temperature while periodically stirring the CRMB with a glass rod. The methodology for the preparation of CRMB is shown schematically in Figure 5.

After the CRMB samples were obtained, the dynamic viscosity was tested with a rotary viscometer. If the dynamic viscosity was less than 3 Pa*s at a test temperature of 135 °C, the basic dependencies of the properties were established. If the viscosity was more than 3 Pa*s, the sample was not used in further tests.

Determination of the influence of formulation and technological factors of rubber modifier on structure parameters and properties of the modified bitumen binder—CRMB (at least three samples are prepared and tested for each percentage of crumb rubber and devulcanizing agent. Standard deviation was not more than 3%), will be carried out by methods specified in the regulatory documents governing the quality of Intertate Standard GOST R 58400.1-2019 [34] (in accordance with AASHTO M 320):

1. to establish the upper limit of the operating temperature range of CRMB (PG X grade) will set the maximum temperature at which CRMB can retain the necessary properties according to the methodology set out in Intertate Standard GOST R 58400.3-2019 [35] (in accordance with AASHTO R 29);
2. the resistance to plastic deformation of CRMB, which contributes to resistance to plastic rutting (in summer and spring-summer periods), will be established by determining the shear stability G*/sin *δ*—an index of bitumen binder’s ability to resist shear effects, determined by the ratio of the complex shear modulus G* to the sine of the phase angle *δ.* Tests of Original Binder and Rolling Thin-Film Oven Residue will be carried out according to the methodology set out in Intertate Standard GOST R 58400.10-2019 [36] (in accordance with AASHTO T 315);
3. the uniformity of the crumb rubber distribution in the volume of the CRMB will be assessed by fluorescence microscopy. Fluorescence microscopy is performed using a MIKMED-2 Luminescence Microscope equipped with a high-pressure mercury ultraviolet lamp. This method is a simple analytical technique for evaluating the morphological characteristics of crumb rubber-modified systems. A small amount of heated sample was loaded and thoroughly crushed between two slides. The slide with the sample was then cooled to room temperature and viewed under a microscope with a magnification of 500× in the MIKMED-2 Luminescence Microscope program.

**Figure 6:**
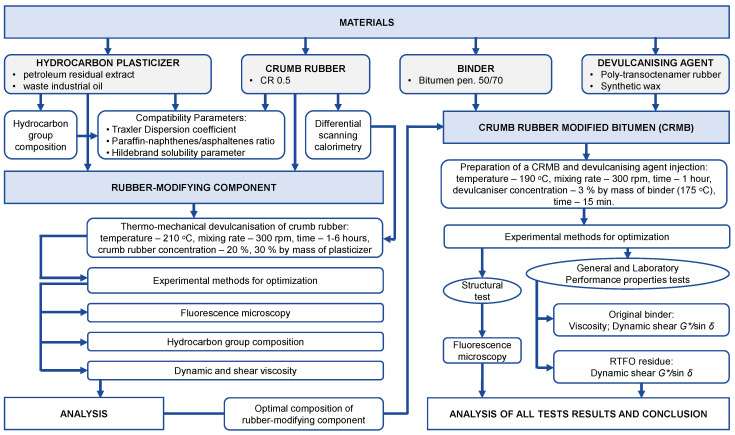
The flow chart of the research approach of this study.

At the same time, it is hoped that the research on this subject can provide additional reference value to the productivity of CRMB. The flow chart of the research approach of this study is shown in Figure 6.

## 3. Results and Discussion

From the analysis of the chromatograms (Figure 3) and the peaks obtained, the group compositions of the hydrocarbon plasticizers were determined, Table 5.

Based on the group hydrocarbon composition of the plasticizers (Table 5), semi-empirical compatibility parameters were calculated for the plasticizers, which include the Traxler dispersion coefficient (Formula (1)), the mass ratio of paraffin-naphthenic compounds to asphaltenes and the Hildebrand solubility parameter (formula (4)). The results of calculating the semi-empirical compatibility parameters for the plasticizers are shown in Table 6. 

An analysis of the plasticizer compatibility data (Table 6) revealed inconsistencies in the semi-empirical parameters, making it difficult to determine the optimum plasticizer based on the calculated values. So, according to the Traxler dispersion coefficient, the most compatible with crumb rubber is petroleum residual extract, whereas according to semi-empirical parameter No. 2, the most compatible is waste industrial oil. According to the Hildebrand solubility parameter, both plasticizers have approximately the same predisposition for compatibility with crumb rubber. Thus, it was found that these semi-empirical parameters need to be refined, but further experimental studies are required.

**Figure 7:**
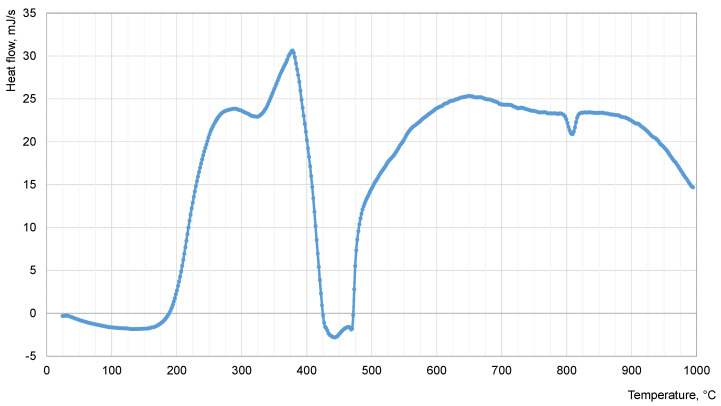
DSC curve for crumb rubber CR 0.5.

Thermal analysis for the crumb rubber was carried out to determine the temperature of the “hydrocarbon plasticizer—crumb rubber” dispersion system. Differential Scanning Calorimetry (DSC) results are shown in Figure 7.

According to the DSC curve for the studied crumb rubber, it can be concluded that the thermo-oxidation of the studied crumb rubber occurs in four stages:

(1) endothermic stage (with heat absorption) at 24–190 °C, associated with evaporation of air moisture and other low molecular weight products;
(2) exothermic stage (with heat release) at 190–425 °C, identified with the main period of thermal oxidation of the material, proceeding up to 378 °C, and the related evaporation of the formed oxidation products, indicating the beginning of the destructive process of rubber granules;
(3) endothermic stage at 425–470 °C with peak at 442 °C characterizes degradation and destruction of rubber granule;
(4) exothermic stage, above 470 °C, characterized by thermal oxidation of crumb degradation products and phase changes of carbon black.

From the results (Figure 7), we can conclude that the degradation of crumb rubber is initiated at 190 °C and proceeds up to 442 °C. However, proceeding from the technical safety of the experiment, it is necessary to consider the flash point of hydrocarbon plasticizers, which for the petroleum residual extract is equal to 284 °C, and for the waste industrial oil—220 °C. Therefore, considering data on DSC for crumb rubber and flash point for plasticizers, we chose the optimum temperature of preparation—210 °C.

**Figure 8:**
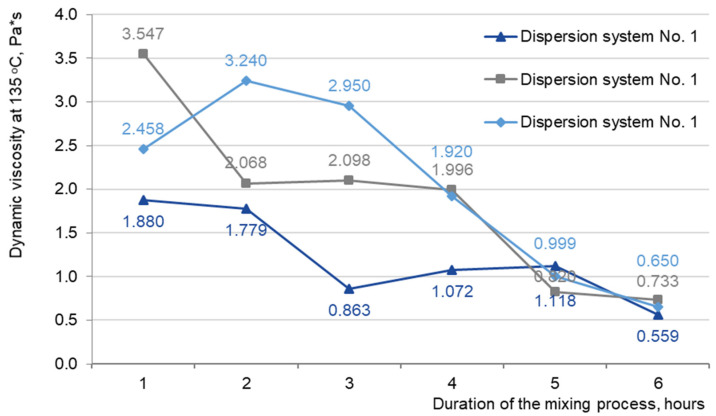
Dynamic viscosity for dispersion system No. 1.

For a choice of the regime of preparation “hydrocarbon plasticizer—crumb rubber” dispersion system and studying the devulcanization process, we have prepared compositions (Table 7) and studied their rheological properties, Figure 8.

**Figure 9:**
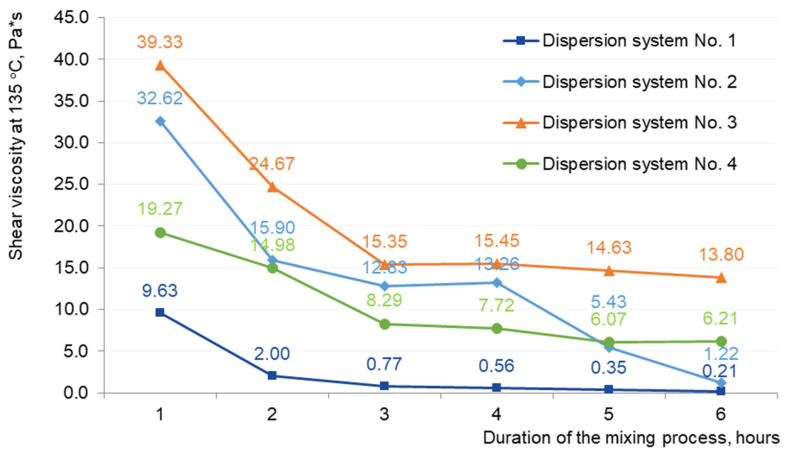
Shear viscosity for dispersion systems No. 1–4.

As shown in Figure 8, one disperse system could not obtain stable results. As you can see, the results are not on one curve. Therefore, the viscosity measurement method on the rotary viscometer is difficult for tested systems. Standard viscosity determination conditions for polymer-modified binders are not suitable for crumb rubber modified. Because of the elastic components, making it difficult to conduct measurements and obtain stable data. And therefore, a significant number of studies are needed to find test conditions. Which a difficult and consistent with existing research in this area [37,38]. So further investigation of the rheological characteristics of disperse systems was carried out by determining the shear viscosity on a dynamic shear rheometer, Figure 9.

The optimal preparation time for all investigated dispersion systems No. 1–4 with different percentages of crumb rubber is when the shear viscosity of dispersion systems reaches approximately the same values. The viscosity curves go to a “plateau”, indicating a maximum degree of swelling of crumb rubber and the formation of structured bonds in the dispersion system [39,40]. The results of shear viscosity measurements (Figure 9) show that as a result of thermo-mechanical action, reaching a “plateau” for all the systems studied occurs after three hours. Further thermomechanical action is not effective. An exception is a sample with a residual extract containing 30% crumb rubber. This is apparently due to the initially lower naphthenic oil content of 22% in the petroleum residual extract compared to 62% in the waste industrial oil. After all, naphthenic oils are the best softeners for rubber, providing a stronger swelling of rubber, their uniform distribution and playing an essential role in improving some of the structural properties of rubber. The high crumb rubber content and low naphthenic oil content led to the delamination of the system after 4 h of thermo-mechanical processing. This is indicated by the shear viscosity values (Figure 9, 4–6 h).

**Figure 10:**
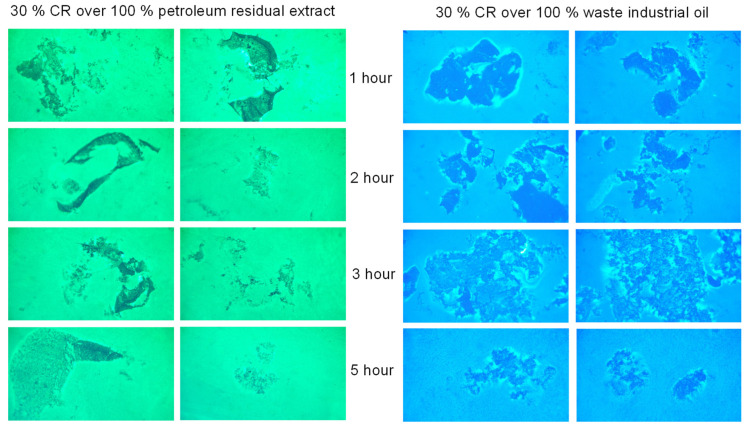
Fluorescence microscopy for dispersion systems No. 2, 4 (200 nm).

For practical applications, the most promising is maximally filled with crumb rubber concentrates of “hydrocarbon plasticizer—crumb rubber” dispersion systems. Therefore, the distribution uniformity was evaluated in systems with 30% crumb rubber. The results are shown in Figure 10.

The analysis of the results obtained, Figure 10, shows that in the sample prepared with waste industrial oil after three hours of thermo-mechanical exposure, a “loosened” structure of the crumb rubber is observed, which indicates the devulcanization process taking place. It consists of partial destruction of the rubber, resulting in local destruction of its spatial structure. That is, the density of the spatial network decreases due to the disintegration of part of the transverse bonds and some of the main molecular chains [39,41]. Further, to stabilize and inhibit the devulcanization process occurring at the stage of CRMB at the final stage of preparation, a devulcanizer/stabilizer is introduced. In the sample prepared with the petroleum residual extract, this effect was not observed. This is also reflected in the lower values obtained for shear viscosity (Figure 9). Therefore, it was not considered further in the study of the dependencies of the CRMB properties.

**Figure 11:**
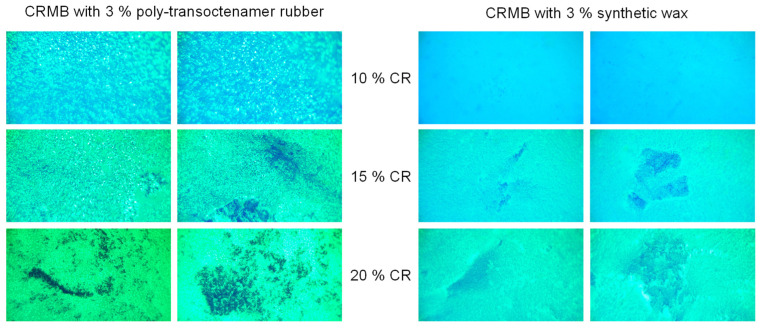
Fluorescence microscopy for CRMB (200 nm).

To establish the features of the influence of crumb rubber on the structure of the CRMB, the compositions were prepared (Table 8), and their performance properties were studied (Figure 11 and Table 9).

Figure 11 shows a heterogeneous system where the polymer-rich phase is associated with swelling or partial degradation of crumb rubber particles in light fractions, such as saturated and aromatic, dispersed in the bitumen matrix as a spherical structure. The size of the polymer phase appears to decrease with optimal curing time and optimal content of crumb rubber. The crumb rubber copolymers are evenly distributed in the bitumen matrix [39]. Analysis of fluorescence microscopy data (Figure 11) reveals that the most uniform distribution of crumb rubber and absence of aggregated particles is observed in CRMB samples with 10% crumb rubber and 3% devulcanizing/stabilising agent (regardless of its variation). It is also worth noting that CRMB samples with 15% and 20% crumb rubber content show visible system delamination, confirmed by structural homogeneity studies, so these samples were not considered further in the study.

According to the test results, CRMB No. 1 and No. 4 meet the viscosity requirements for PG bitumen, their viscosity being less than 3 Pa*s. The high-temperature value of Performance Grade for CRMB No.1 prepared with poly-transoctenamer rubber is higher than that of CRMB No.4. However, it is worth noting that for CRMB No. 4 prepared with 3% synthetic wax, the Dynamic shear for Original Binder and Rolling Thin-Film Oven Residue corresponds to the same high-temperature value of Performance Grade. This demonstrates a stable structure and stabilization of the devulcanization process, which makes it promising to investigate further the effect of this synthetic wax on CRMB properties.

It is worth mentioning that scientific research is currently ongoing to adjust and optimise compositions of CRMB, select a harder bitumen base and develop technological bases to produce more concentrated rubber-containing dispersion systems to increase the operating temperature interval of the CRMB produced.

## 4. Conclusions

The semi-empirical Traxler and Hildebrand compatibility parameters are unreliable for “hydrocarbon plasticizer—crumb rubber” dispersion systems. It is found that the most suitable semi-empirical coefficient for establishing a compatible plasticizer to crumb rubber is one based on the ratio of paraffin-naphthenic compounds to asphaltene-resin compounds.

Using differential scanning calorimetry, the temperature regimes of the degradation of crumb rubber were investigated. It was found that the degradation of crumb rubber is initiated at 190 °C and proceeds up to 442 °C. Considering the technical safety of the experiment and values of the flash point of hydrocarbon plasticizers, an optimum preparation temperature of 210 °C has been established.

When selecting the methods of investigation of the “hydrocarbon plasticizer—crumb rubber” dispersion system, it was found that the method of determining the dynamic viscosity of the dispersion system on a rotary viscometer is not applicable due to the presence of elastic components, making it difficult to conduct measurements and obtain reliable data. The most suitable method is the shear viscosity method.

The hydrocarbon plasticizer with high naphthenic oil content was found to be the most compatible with crumb rubber. To study the possibility of providing stabilization and inhibition of the ongoing devulcanization process of crumb rubber, six samples of CRMB with devulcanizing/stabilizing agents were prepared and examined. Based on the results of fluorescent microscopy, it was found that the most uniform distribution of crumb rubber and lack of aggregated particles is observed in the samples of CRMB with 10% of crumb rubber and 3% devulcanizing/stabilizing agent, regardless of its variety. The CRMB samples with a higher crumb content of 15% and 20% and with synthetic wax have been found to exhibit visible system delamination.

The test results of the CRMB obtained have shown that the viscosity of the systems meets the requirements of the standard for PG bitumen. Synthetic wax as devulcanizing/stabilising agent for crumb rubber in the binder volume is more promising than poly-transoctenamer rubber.

## Figures and Tables

**Table 1:** Physical properties of crumb rubber CR 0.5.

Name of Parameter	Actual Value
Mass fraction of rubber sifted through the sieve, % with mesh No. 0.5 with mesh No. 0.63	98.18 100
Mass fraction of cord residue, %	0.77
Mass loss on drying, %	0.34
Mass fraction of ferrous metal particles, %	traces
Mass fraction of mechanical impurities in base metal, stones, glass, etc.	absence
The presence of lumps of fluffed cord fiber	absence

**Table 2:** Physical and chemical properties of the petroleum residual extract.

Name of Parameter	Actual Value
Kinematic viscosity at 50 °C, mm^2^/s	905.01
Kinematic viscosity at 100 °C, mm^2^/s	47.69
Flashpoint, °C	284.3

**Table 3:** Physical and chemical properties of waste industrial oil.

Name of Parameter	Actual Value
Kinematic viscosity at 50 °C, mm^2^/s	21.88
Kinematic viscosity at 100 °C, mm^2^/s	5.33
Flashpoint, °C	220.5

**Table 4:** Physical and chemical properties of bitumen BND 50/70.

Name of Parameter	Requirements of Interstate Standard GOST 33133 [33]	Actual Value
Penetration at 25 °C, 0.1 mm	51–70	55
Penetration at 0 °C, 0.1 mm	≥18	23
Softening point (Ring and ball), °C	≥51	53
Ductility at 0 °C, cm	≥3.5	3.5
Fraass breaking point, °C	≤−16	−22
Flashpoint, °C	≥230	248
Weight change after aging, %	≤0.6	0.27
Softening point change after aging, %	≤7	5.2

**Table 5:** Hydrocarbon group composition of the plasticizers tested.

Name of Hydrocarbon Groups	Hydrocarbon Group Content in Petroleum Residual Extract, %	Hydrocarbon Group Content in Waste Industrial Oil, %
Oils containing:		
paraffin-naphthenic	22.6	62.7
aromatic containing:		
light aromatics	14.6	12.9
medium aromatics	13.9	4.0
heavy aromatics	24.6	3.2
resins containing:		
resins I	5.3	3.2
resins II	10.6	7.0
asphaltenes	8.4	7.0

**Table 6:** Semi-empirical plasticizer compatibility parameters.

No.	Calculated Semi-Empirical Parameters for Plasticizers	Petroleum Residual Extract	Waste Industrial Oil
1	Traxler dispersion coefficient	2.2	0.4
2	The mass ratio of paraffin-naphthenic compounds to asphaltenes	2.7	9.0
3	Hildebrand solubility parameter for plasticizer, (J/cm^3^)^0.5^	25.7	24.2
	Hildebrand solubility parameter for modifier (crumb rubber) *, (J/cm^3^)^0.5^	16.8	
	Δδ, (J/cm^3^)^0.5^	8.9	7.4

* According to the crumb rubber manufacturer, more than 60% of the crumb rubber contains isoprene rubber, so the solubility parameter for the crumb rubber modifier was calculated for isoprene rubber.

**Table 7:** Compositions of “hydrocarbon plasticizer—crumb rubber” dispersion systems.

System Number	Dispersion System Component, %		
	Petroleum Residual Extract	Waste Industrial Oil	Crumb Rubber CR 0.5
1	100	-	20
2	100	-	30
3	-	100	20
4	-	100	30

**Table 8:** Compositions of crumb rubber modified binders.

CRMB Number	CRMB Component, %						Homogeneity of CRMB
	Bitumen pen. 50/70	Petroleum Residual Extract	Waste Industrial Oil	Crumb Rubber CR 0.5	Poly-TransoctenamerRubber (over 100%)	Synthetic Wax (over 100%)	
1	56.6	33.4	10	3	-	56.6	yes
2	34.8	50.2	15	3	-	34.8	yes
3	13.2	66.8	20	3	-	13.2	yes
4	56.6	33.4	10	-	3	56.6	yes
5	34.8	50.2	15	-	3	34.8	no
6	13.2	66.8	20	-	3	13.2	no

**Table 9:** Operational properties of crumb rubber modified bitumen.

Name of Parameter	CRMB No. 1	CRMB No. 4	Test Methods
Original Binder			
Viscosity: max 3 Pa*s, test temp 135 °C, Pa*s	0.22	0.08	Interstate Standard GOST 33137 [42]
Dynamic shear: G*/sin *δ*, min 1.00 kPa test temp at 10 rad/s, °C	58	34	Interstate Standard GOST R 58400.10 [36]
Continuous Grading Temperature, °C	61.3	38.8	Interstate Standard GOST R 58400.10 [36]
Rolling Thin-Film Oven Residue			
Dynamic shear: G*/sin *δ*, min 2.20 kPa test temp at 10 rad/s, °C	46	34	Interstate Standards GOST 33140 [43], GOST R 58400.10 [36]
Continuous Grading Temperature, °C	47.3	39.0	Interstate Standards GOST 33140 [43], GOST R 58400.10 [36]

## Data Availability

Data are contained within the article.

## References

[1] Ali H., Hasan H.J. (2021). Assessment the performance of asphalt mixtures modified with waste tire rubber at high temperatures. J. Phys. Conf. Ser..

[2] Wang X., Hong L., Wu H., Liu H., Jia D. (2021). Grafting waste rubber powder and its application in asphalt. Constr. Build. Mater..

[3] Shen J., Amirkhanian S., Xiao F., Tang B. (2009). Influence of surface area and size of crumb rubber on high temperature properties of crumb rubber modified binders. Constr. Build. Mater..

[4] Yu J., Ren Z., Yu H., Wang D., Svetlana S., Korolev E., Gao Z., Guo F. (2018). Modification of Asphalt Rubber with Nanoclay towards Enhanced Storage Stability. Materials.

[5] Yu H., Chen Y., Wu Q., Zhang L., Zhang Z., Zhang J. (2020). Decision support for selecting optimal method of recycling waste tire rubber into wax-based warm mix asphalt based on fuzzy comprehensive evaluation. J. Clean. Prod..

[6] Duan H., Zhu C., Li Y., Zhang H., Zhang S., Xiao F., Amirkhanian S. (2021). Effect of crumb rubber percentages and bitumen sources on high-temperature rheological properties of less smell crumb rubber modified bitumen. Constr. Build. Mater..

[7] Xiao M., Jitao Y., Chen L. (2021). Progress in combined modification of rubberized asphalt. IOP Conf. Series Earth Environ. Sci..

[8] Li J., Chen Z., Xiao F., Amirkhanian S.N. (2021). Surface activation of scrap tire crumb rubber to improve compatibility of rubberized asphalt. Resour. Conserv. Recycl..

[9] Wang H., Apostolidis P., Zhu J., Liua X., Skarpas A., Erkens S. (2020). The role of thermodynamics and kinetics in rubber–bitumen systems: A theoretical overview. Int. J. Pavement Eng..

[10] Bressi S., Fiorentini N., Huang J., Losa M. (2019). Crumb Rubber Modifier in Road Asphalt Pavements: State of the Art and Statistics. Coatings.

[11] Makoundou C., Johansson K., Wallqvist V., Sangiorgi C. (2021). Functionalization of Crumb Rubber Surface for the Incorporation into Asphalt Layers of Reduced Stiffness: An Overview of Existing Treatment Approaches. Recycling.

[12] Sheng Y., Li H., Geng J., Tian Y., Li Z., Xiong R. (2017). Production and performance of desulfurized rubber asphalt binder. Int. J. Pavement Res. Technol..

[13] Li H., Dong B., Zhao D. (2019). Physical, Rheological and Stability Properties of Desulfurized Rubber Asphalt and Crumb Rubber Asphalt. Arab. J. Sci. Eng..

[14] Li X., Xu X., Liu Z. (2020). Cryogenic grinding performance of scrap tire rubber by devulcanization treatment with ScCO2. Powder Technol..

[15] Zheng W., Wang H., Chen Y., Ji J., You Z., Zhang Y. (2021). A review on compatibility between crumb rubber and asphalt binder. Constr. Build. Mater..

[16] Radeef H.R., Hassan N.A., Abidin A.R.Z., Mahmud M.Z.H., Yaacob H., Mashros N., Mohamed A. (2021). Effect of aging and moisture damage on the cracking resistance of rubberized asphalt mixture. Mater. Today Proc..

[17] Wang T., Xiao F., Amirkhanian S., Huang W., Zheng M. (2017). A review on low temperature performances of rubberized asphalt materials. Constr. Build. Mater..

[18] Qurashi I.A., Swamy A.K. (2018). Viscoelastic properties of recycled asphalt binder containing waste engine oil. J. Clean. Prod..

[19] Cai J., Song C., Zhou B., Tian Y., Li R., Zhang J., Pei J. (2019). Investigation on high-viscosity asphalt binder for permeable asphalt concrete with waste materials. J. Clean. Prod..

[20] Ren S., Liu X., Lin P., Wang H., Fan W., Erkens S. (2021). The continuous swelling-degradation behaviors and chemo-rheological properties of waste crumb rubber modified bitumen considering the effect of rubber size. Constr. Build. Mater..

[21] Wang H., Liu X., Erkens S., Skarpas A. (2020). Experimental characterization of storage stability of crumb rubber modified bitumen with warm-mix additives. Constr. Build. Mater..

[22] Guo F., Zhang J., Pei J., Zhou B., Falchetto A.C., Hu Z. (2020). Investigating the interaction behavior between asphalt binder and rubber in rubber asphalt by molecular dynamics simulation. Constr. Build. Mater..

[23] Wang H., Liu X., Apostolidis P., Erkens S., Skarpas A. (2020). Experimental Investigation of Rubber Swelling in Bitumen. Transp. Res. Rec..

[24] Xu X., Leng Z., Lan J., Li R., Tan Z., Sreeram A., Di Benedetto H., Baaj H., Chailleux E., Tebaldi G., Sauzéat C., Mangiafico S. (2022). Rubber-Bitumen Interaction of Plant-Blended Rubberized Bitumen Prepared Under Various Blending Conditions. Proceedings of the RILEM International Symposium on Bituminous Materials.

[25] Lv Q., Huang W., Zheng M., Hu Y., Yan C., Wang J. (2022). Understanding the particle effects and interaction effects of crumb rubber modified asphalt regarding bonding properties. Constr. Build. Mater..

[26] Mahmoudi Y., Mangiafico S., Sauzéat C., Di Benedetto H., Pouget S., Loup F., Faure J.-P., Di Benedetto H., Baaj H., Chailleux E., Tebaldi G., Sauzéat C., Mangiafico S. (2022). Experimental Evaluation of Swelling and Absorption of Crumb Rubber Aggregates. Proceedings of the RILEM International Symposium on Bituminous Materials.

[27] Lanotte M. (2022). Soft Computing Approach for Predicting the Effects of Waste Rubber–Bitumen Interaction Phenomena on the Viscosity of Rubberized Bitumen. Sustainability.

[28] Rochlani M., Leischner S., Wareham D., Caro S., Falla G.C., Wellner F. (2022). Investigating the performance-related properties of crumb rubber modified bitumen using rheology-based tests. Int. J. Pavement Eng..

[29] Petersen J.C. (2000). Chapter 14 Chemical Composition of Asphalt as Related to Asphalt Durability. Dev. Pet. Sci..

[30] Wang C., Xie T., Cao W. (2019). Performance of bio-oil modified paving asphalt: Chemical and rheological characterization. Mater. Struct..

[31] Yen N.T.T. (2021). Development of Scientific and Technological Foundations for the Production of Rubber-Containing Road Binders. Ph.D. Thesis.

[32] Gallu R., Mechin F., Dalmas F., Gerard J.F., Perrin R., Loup F. (2020). On the use of solubility parameters to investigate phase separation-morphology-mechanical behavior relationships of TPU. Polymer.

[33] (2014). Automobile Roads of General Use. Viscous Road Petroleum Bitumens.

[34] (2019). Automobile Roads of General Use. Petroleum-Based Bitumen Binder. Specifications Based on Operational Temperature Range.

[35] (2019). Automobile Roads of General use. Petroleum-Based Bitumen Binders. The Procedure for Determination of the Brand.

[36] (2019). Automobile Roads of General Use. Petroleum-Based Bitumen Binders. Method for Determination of the Properties Using a Dynamic Shear Rheometer (DSR).

[37] Ding Z., Li P., Zhang J., Bing H., Yue X. (2020). Analysis of viscosity test conditions for crumb-rubber-modified asphalt. Constr. Build. Mater..

[38] Zoorob S.E., Castro-Gomes J.P., Oliveira L.A.P. (2012). Assessing low shear viscosity as the new bitumen Softening Point test. Constr. Build. Mater..

[39] Xing B., Feng Y., Sun S., Qian C., Fang C., Lv X., Song A., Lyu Y. (2023). Investigations on the rheological and swelling-degradation behavior of crumb rubber within the bituminous matrix. Constr. Build. Mater..

[40] Dong D., Huang X., Li X., Zhang L. (2012). Swelling process of rubber in asphalt and its effect on the structure and properties of rubber and asphalt. Constr. Build. Mater..

[41] Liang M., Xin X., Fan W., Sun H., Yao Y., Xing B. (2015). Viscous properties, storage stability and their relationships with microstructure of tire scrap rubber modified asphalt. Constr. Build. Mater..

[42] (2014). Automobile Roads of General Use. Viscous Road Petroleum Bitumens. Method for Determining Dynamic Viscosity with a Rotational Viscometer.

[43] (2014). Automobile Roads of General Use. Viscous Road Petroleum Bitumens. High Temperature and Air Aging Method (RTFOT Method).

